# Antecedent infections in Guillain‐Barré syndrome: a single‐center, prospective study

**DOI:** 10.1002/acn3.50946

**Published:** 2019-11-12

**Authors:** Yanlei Hao, Weifang Wang, Bart C. Jacobs, Baojun Qiao, Mengshi Chen, Daiqiang Liu, Xungang Feng, Yuzhong Wang

**Affiliations:** ^1^ Department of Neurology Affiliated Hospital of Jining Medical University Jining Shandong Province China; ^2^ Department of Neurology and Immunology Erasmus University Medical Centre Rotterdam The Netherlands; ^3^ Department of Epidemiology and Health Statistics School of Public Health Central South University Changsha Hunan Province China; ^4^ Central Laboratory Affiliated Hospital of Jining Medical University Jining Shandong Province China

## Abstract

**Objective:**

To investigate the spectrum of antecedent infections in Chinese patients with Guillain‐Barré syndrome (GBS) and analyze the infections‐related clinical phenotypes locally.

**Methods:**

A prospective case‐control study of 150 patients diagnosed with GBS and age‐ and sex‐matched neurological and healthy controls was performed to investigate recent infections of 14 pathogens serologically and collect the clinical data during a follow‐up of 12 months.

**Results:**

In total, 53% of patients with GBS had a positive serology for recent infection, including *Campylobacter jejuni* (27%), influenza A (17%) and B (16%), hepatitis A virus (5%), dengue virus (3%), cytomegalovirus (3%), Epstein–Barr virus (3%), *Mycoplasma pneumoniae* (2%), herpes simplex virus (2%), varicella‐zoster virus (1%), and rubella virus (1%). Serology for infections of hepatitis E virus, *Haemophilus influenzae*, and Zika virus was negative. There was a higher frequency of *C. jejuni*, influenza A, influenza B, and hepatitis A virus infections in GBS patients than both the neurological and healthy controls. *C. jejuni* infection was more frequent in younger GBS patients and was associated with antibodies against GM1, GalNAc‐GD1a, and GM1:galactocerebroside complex. Influenza B infection was associated with a pure motor form of GBS.

**Interpretation:**

*C. jejuni*, influenza A, influenza B, and hepatitis A virus serve as the most common cause of antecedent infections in GBS locally. Influenza B‐related GBS may represent a pure motor phenotype. Differences in the infectious spectrum worldwide may contribute to the geographical clinical heterogeneity of GBS.

## Introduction

Guillain‐Barré syndrome (GBS) is an immune‐mediated polyradiculoneuropathy characterized by a rapidly progressive flaccid paresis. Recent evidence supports GBS as a spectrum disorder with regional variation and significant heterogeneity including clinical presentation, electrophysiology, and outcome.^1^, ^2^ Two thirds of the patients complained of antecedent infections before the onset of neurological signs.^3^ Some antecedent infections were associated with various clinical phenotypes in GBS. Typically, *Campylobacter jejuni* bearing the gangliosides‐like lipo‐oligosaccharides (LOS) accounts for the pathogenesis of axonal GBS, particularly acute motor axonal neuropathy.^4^ Cytomegalovirus (CMV) infection is associated with severe motor sensory deficits, demyelination, and antibodies to the ganglioside GM2.^3^ *Mycoplasma pneumoniae* infection is associated with anti‐galactocerebroside (GalC) antibodies and pediatric GBS.^5^ Global variation in infection burden may at least in part explain the regional differences in clinical presentation and subtype of GBS. In the 1990s, a study from Northern China reported axonal GBS as the major subtype in China associated with a high frequency of *C. jejuni* infection.^6^ More recent studies, however, showed that currently demyelinating GBS was the predominant subtype in both Northeastern and Southern China.^7^, ^8^ The rapid changes in the socioeconomic status of China may have influenced the exposure to infections and resulted in a shift of the predominant GBS subtype. Furthermore, many GBS patients developed liver dysfunction before treatment without obvious causes, that may be related to specific types of antecedent infections.^9^ The current study aimed to investigate the spectrum of GBS‐related antecedent infections in a Chinese local area and analyze the infection‐related clinical features.

## Methods

### Patients and blood samples

This study was performed in the Affiliated Hospital of Jining Medical University, a central hospital regionally in Southwest of Shandong Province, Northern China, where it has a population of 17.1 million with an urban–rural ratio of 1.19. Written informed consent was obtained from all participants, and study procedures were approved by the local Ethics Committee (reference 2013B017 and 2016B006). From October 2013 to June 2017, a total of 150 consecutive patients meeting the diagnostic criteria for GBS and its variants^10^, ^11^ from the Affiliated Hospital of Jining Medical University were included in this study, of whom 19 also participated in the International GBS Outcome Study.^12^ For each participant, the pretreatment serum was collected and kept at −80°C until use. The clinical data include: age, sex, upper respiratory tract infection or gastrointestinal infection within 4 weeks before developing neurological signs, motor and sensory deficits, cranial nerve involvement, ataxia, tendon reflex, pain, mechanical ventilation, nerve conduction study (NCS) within 2 weeks after onset,^13^ albuminocytological dissociation in cerebrospinal fluid (CSF), and GBS disability score (GBS‐DS)^14^ at nadir and 12 months. The disability score at 12 months was obtained from 146 (97%) of the patients by a telephone follow‐up or outpatient revisit. No follow‐up NCS was performed for the patients. Four patients were lost to follow‐up. To explore the relation between antecedent infection and liver function, data of liver function tests from patients before treatment were also collected. For the study of pretreatment liver dysfunction, 18 of the patients were excluded because of one or more of the reasons below: with a previous diagnosis of liver diseases, alcohol abuse or recent intake of liver‐toxic or liver enzyme‐inducing drugs, with definite factors resulting in muscle damage, and elevation of transaminases. The liver dysfunction was defined as either alanine aminotransferase or aspartate aminotransferase becoming 1.5 times higher than the upper limit of normal values.

### Controls

After the inclusion of each patient with GBS, a sex‐ and age‐matched inpatient with other neurological diseases (OND) and sex‐ and age‐matched healthy donors in the same period were, respectively, selected from the local biological sample bank of the hospital established from July 2012. Among the 150 OND controls were included patients with a cerebral infarction (*n* = 23), cerebral hemorrhage (*n* = 27), peripheral vertigo (*n* = 28), Bell's palsy (*n* = 39), migraine (*n* = 15), myasthenia gravis (*n* = 4), meningitis (*n* = 3), Parkinson disease (*n* = 6), and epilepsy (*n* = 5). The 150 healthy controls were recruited from the same hospital and in all heathy controls, organic diseases were ruled out by the routine medical examination.

### Antecedent infectious spectrum detection

A total of 14 infectious agents were selected according to previous report^3^ and unpublished data from Department of Infectious Diseases, Chinese Centre for Disease Control, including *C. jejuni*, *M. pneumoniae*, *Haemophilus influenzae*, influenza A and B virus, herpes simplex virus, varicella‐zoster virus, dengue virus, rubella virus, CMV, Epstein–Barr virus (EBV), hepatitis A virus, hepatitis E virus, and Zika virus. The details of ELISA kits for detecting the infectious agents are shown in Table S1. The serum samples were tested according to the manufactory instructions. To detect IgM antibodies, the serum was pretreated to remove the IgG antibodies and prevent false positivity. *C. jejuni*, influenza A, and influenza B virus infections were defined as the presence of IgA and/or IgM antibodies. *H. influenzae* infection was defined as the presence of IgG antibodies. Infections of *M. pneumoniae*, hepatitis A virus, herpes simplex virus, varicella‐zoster virus, EBV, dengue virus, rubella virus, CMV, hepatitis E virus, and Zika virus were defined as the presence of IgM antibodies. The serology for specific infections in the controls was detected only if more than five GBS patients were positive for this infection as previously described.^3^

### Anti‐glycolipid antibody assay

The serum samples of patients with GBS were tested in duplicate for IgM and IgG antibodies against GM1, GM2, GM3, GM1b, GD1a, N‐acetylgalactosaminyl GD1a (GalNAc‐GD1a), GD1b, GT1a, GQ1b, GalC, sulfatide, GM1:GalC, GM1:sulfatide, GalC:cholesterol, and GalC:sulfatide complexes as previously described.^15^ In general, a vinyl ELISA plate (Corning, ME, USA) was coated with the glycolipid or glycolipid complex (5 pmol per well; for glycolipid complex, 2.5 pmol each). The serum diluted in phosphate buffered saline (PBS) (0.1 M, pH 7.4) containing 0.5% casein (1:500) was added into the plates for incubation overnight at 4°C. After three times of washing with 0.1 M PBS containing 0.05% Tween 20, the plates were added with horseradish peroxidase‐conjugated goat anti‐human IgG (gamma chain) (Thermo Scientific, Rockford, IL, USA) (1:3000 in PBS containing 0.5% casein) or horseradish peroxidase‐conjugated goat anti‐human IgM (heavy chain) (Thermo Scientific) (1:10000) and incubated at 37°C for 1 h. After washing the plates with the same washing buffer, the binding of IgG or IgM antibodies was visualized by O‐phenylenediamine (Sigma, MO, USA) developing solution in the darkness for 15 min and then the reaction was stopped by 2N hydrochloride. The absorbance at 492 nm/630 nm (as reference) was measured using a ChroMate® Microplate Reader (Awareness Technology, Palm City, USA). Each sample was tested with a blank and negative control for quality control. With reference to a blank control, the optical density value over 0.1 was considered positive. A positive serology for anti‐glycolipid antibodies was defined as the presence of either IgG or IgM or both.

### Data availability

Our data will be shared by request from any qualified investigator for scientific purposes.

### Statistical analysis

Normally distributed continuous data were presented as means and standard deviations. The categorical variables were shown as n (%). The difference in the frequency of infections in patients with GBS and the controls was compared by the Chi‐square test or Fisher's exact test. For patients with and without infections, the difference in demographic and clinical features, anti‐glycolipid antibodies, and clinical subtypes were compared by Chi‐square test or Fisher's exact test. The analysis was performed with the SPSS 20.0 analysis software (IBM, Armonk, NY). A two‐sided *P* < 0.05 was considered to be significant.

## Results

### Antecedent infectious spectrum in patients with GBS

**Figure 1:**
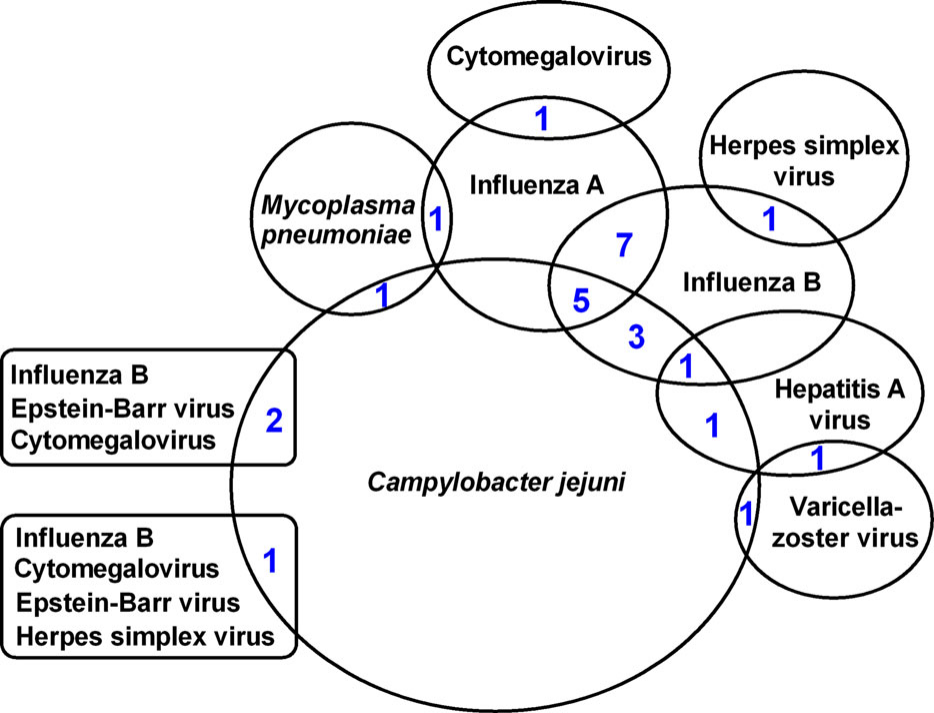
The number of patients with more than one infection.

The demographic and clinical features of the patients with GBS are shown in Table 1. There was no difference in age or sex between the patients with GBS and the controls. The mean age of patients with GBS was 51.0; interquartile range (IQR) was 41 to 64 and the male to female ratio was 1.2 (81/69). For patients with OND and the healthy controls, the mean age was 51.9 (IQR, 42–64) and 51.3 (IQR, 42–64), respectively; the sex ratio was both the same 1.2 (81/69). Of the 150 patients with GBS, 53% (80/150) had positive serology for either *C. jejuni* (*n* = 40, 27%), influenza A (*n* = 26, 17%), influenza B (*n* = 24, 16%), hepatitis A virus (*n* = 7, 5%), dengue virus (*n* = 4, 3%), CMV (*n* = 4, 3%), EBV (*n* = 4, 3%), *M. pneumoniae* (*n* = 3, 2%), herpes simplex virus (*n* = 3, 2%), varicella‐zoster virus (*n* = 2, 1%), and rubella virus (*n* = 1, 1%). There were significant higher frequencies of *C. jejuni*, influenza A, influenza B, and hepatitis A virus infections in patients with GBS than in the controls (Table 2). Twenty‐six (17%) patients had more than one infection (Fig. 1). Eighty percent of patients (120/150) were from rural areas. There was no difference in the frequency of antecedent infections in patients with GBS between urban and rural areas (Table S2). None of the patients had positive serology for hepatitis E virus, *H. influenza*, and Zika virus.

**Table 1:** Demographic and clinical characteristics of 150 patients with Guillain‐Barré syndrome.

Characteristic	*n* (%)
Age, mean (standard deviation)	51.0 (16.1)
Male/female ratio	1.2 (81/69)
Antecedent infection within 4 weeks	
Upper respiratory tract infection	36 (24)
Gastrointestinal infection	19 (13)
Motor deficits	
Upper and lower limb weakness	111 (74)
Upper limb weakness only	5 (3)
Lower limb weakness only	11 (7)
None	23 (15)
Sensory deficits	62 (41)
Cranial nerve involvement	
Oculomotor weakness	23 (15)
Facial weakness	30 (20)
Bulbar weakness	29 (19)
None	68 (45)
Ataxia	6 (4)
Tendon reflex at the nadir	
Hyporeflexia or areflexia	128 (85)
Normal	22 (15)
Pain	13 (9)
Disability score at the nadir	
1	35 (23)
2	31 (21)
3	20 (13)
4	45 (30)
5	17 (11)
6	2 (1)
Albuminocytological dissociation in CSF	95/123 (77)
Single nerve conduction study	
Primary demyelinating	41/120 (34)
Primary axonal	35/120 (29)
Unclassified	27/120 (23)
Normal	17/120 (14)
Disability score at 12 months	
0–1	113/146 (77)
2	13/146 (9)
3	8/146 (6)
4	5/146 (3)
6	7/146 (5)
Pretreatment liver dysfunction	13 (17/132)

CSF, cerebrospinal fluid.

**Table 2:** Frequency of antecedent infections in patients with Guillain‐Barré syndrome and the controls.

	GBS (*n* = 150)	OND controls (*n* = 150)	OR (95% CI)	*P* value	HC (*n* = 150)	OR (95% CI)	*P* value
No infection	70 (47)	128 (85)	Reference		132 (88)	Reference	
*Campylobacter jejuni*	40 (27)	10 (7)	7.3 (3.5, 15.5)	<0.001	13 (9)	5.8 (2.9, 11. 6)	<0.001
Influenza A	26 (17)	8 (5)	5.9 (2.6, 13.8)	<0.001	10 (7)	4.9 (2.2, 10.7)	<0.001
Influenza B	24 (16)	7 (5)	6.3 (2.6, 15.3)	<0.001	9 (6)	5.0 (2.2, 11.4)	<0.001
Hepatitis A virus	7 (5)	3 (2)	4.3 (1.1, 17.0)	0.027	0 (0)	‐	‐
Dengue virus	4 (3)						
Cytomegalovirus	4 (3)						
Epstein–Barr virus	4 (3)						
*Mycoplasma pneumoniae*	3 (2)						
Herpes simplex virus	3 (2)						
Varicella‐zoster virus	2 (1)						
Rubella virus	1 (1)						

The data are shown as *n* (%).

OR, odds ratio; CI, confidence interval; GBS, Guillain‐Barré syndrome; OND, other neurological disease; HC, healthy controls. No infection of the hepatitis E virus, *Haemophilus influenzae*, and Zika virus was detected.

### Infection‐related clinical features

As shown in Table 3, the comparison was made among the GBS patients with infection of *C. jejuni*, influenza A, and influenza B as well as patients with no infections. There were significantly younger age and higher frequency of antecedent diarrhea complaints in patients with *C. jejuni* infection than the controls. All seven patients with preceding influenza B infection had a pure motor GBS without sensory deficits, which differed from the patients with infection of *C. jejuni*, influenza A, and patients without infection (*P* = 0.037). In addition, these patients with influenza B infection had no ataxia (0/7), no pain (0/7), no demyelinating subtypes (0/5) and high frequency of mechanical ventilation (2/7, 29%), and pretreatment liver dysfunction (2/7, 29%). There was no mechanical ventilation (0/11) in GBS patients following influenza A infection. There was no pretreatment liver dysfunction (0/25) in GBS patients following *C. jejuni* infection. With regard to patients with *C. jejuni*, influenza A and B virus as well as without infection, there was no difference in frequency of patients on cranial nerve involvement, ataxia, tendon reflex at nadir, pain, mechanical ventilation, electrophysiological classification, and pretreatment liver dysfunction.

**Table 3:** Antecedent infections and clinical characteristics of patients with Guillain‐Barré syndrome.

Characteristic	No infection (*n* = 70)	*Campylobacter jejuni* (*n* = 25)	Influenza A (*n* = 11)	Influenza B (*n* = 7)	*P* value*
Age, mean (standard deviation)	51.8 (15.3)	44.6 (15.3)	60.6 (10.3)	54.3 (19.3)	0.03
Antecedent infection within 4 weeks					
Upper respiratory tract infection	16 (23)	7 (28)	3 (27)	2 (28)	0.032
Gastrointestinal infection	6 (9)	8 (32)	0 (0)	0 (0)	
None	48 (68)	10 (40)	8 (73)	5 (72)	
Motor deficits	61 (87)	21 (84)	9 (82)	6 (86)	0.969
Sensory deficits	35 (50)	8 (32)	6 (55)	0 (0)	0.037
Cranial nerve involvement	41 (59)	15 (60)	6 (55)	6 (86)	0.766
Ataxia	4 (6)	1 (4)	1 (9)	0 (0)	0.846
Hyporeflexia or areflexia at nadir	62 (89)	24 (96)	9 (82)	5 (71)	0.276
Pain	3 (4)	3 (12)	2 (18)	0 (0)	0.227
Mechanical ventilation	9 (13)	3 (12)	0 (0)	2 (29)	0.353
Disability score at nadir					
˂4	35 (50)	13 (52)	8 (73)	3 (43)	0.524
≥4	35 (50)	12 (48)	3 (27)	4 (57)	
Albuminocytological dissociation in CSF	41/58 (71)	18/20 (90)	9/9 (100)	5/5 (100)	0.053
Single nerve conduction study					
Primary demyelinating	21/57 (37)	5/17 (29)	3/9 (33)	0/5 (0)	0.661
Primary axonal	14/57 (25)	7/17 (41)	2/9 (22)	2/5 (40)	
Unclassified	13/57 (23)	2/17 (12)	3/9 (33)	1/5 (20)	
Normal	9/57 (16)	3/17 (18)	1/9 (11)	2/5 (40)	
Disability score at 12 months					
˂2	52/68 (76)	18 (72)	9/10 (90)	5 (71)	0.708
≥2	16/68 (24)	7 (28)	1/10 (10)	2 (29)	
Pretreatment liver dysfunction	12/63 (19)	0/25 (0)	2/11 (18)	2/7 (29)	0.109

CSF, cerebrospinal fluid. If not specified, the data are shown as n (%).

* The *P* value showed the difference among the four groups.

### Antecedent infections and the clinical variants

**Figure 2:**
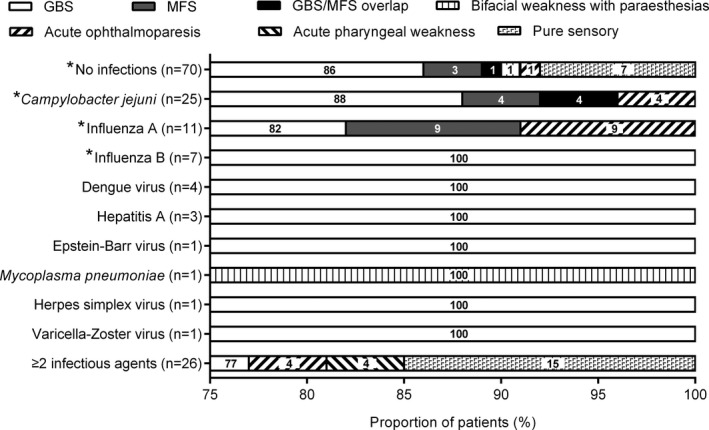
Antecedent infections and the clinical variants of Guillain‐Barré syndrome. As shown, Guillain‐Barré syndrome (GBS) constitutes the major subtypes in all of the patients with and without infections except that one patient with *Mycoplasma pneumoniae* had the bifacial weakness with paraesthesias. *There was no difference in frequency of GBS among the patients with and without infections (partition of Chi‐square test, *P* = 0.607). MFS, Miller Fisher syndrome.

The patients were classified into GBS (*n* = 125, 83%), Miller Fisher syndrome (MFS) (*n* = 4, 3%), acute ophthalmoparesis (*n* = 4, 3%), GBS/MFS overlap (*n* = 2, 1%), bifacial weakness with paraesthesias (*n* = 2, 1%), acute pharyngeal weakness (*n* = 2, 1%), and pure sensory subtype (*n* = 11, 7%). As shown in Figure 2, there was no association between these variants of GBS and the presence of specific types of preceding infection.

### Antecedent infections and anti‐glycolipid antibodies

As shown in Table S3, the frequency of antibodies against glycolipids and glycolipids complex in 150 of patients with GBS was anti‐GM1 (39%), anti‐GM1b (1%), anti‐GD1a (11%), anti‐GD1b (19%), anti‐GalNAc‐GD1a (11%), anti‐GQ1b (13%), anti‐GM1:GalC complex (23%), anti‐GM1:sulfatide complex (20%), and anti‐GalC:sulfatide complex (1%), respectively. There was significantly higher frequency of antibodies against GM1, GalNAc‐GD1a, and GM1:GalC complex in patients following *C. jejuni* infection than patients without infections (*P* = 0.002, *P* < 0.001, and *P* = 0.019, respectively)*.* The anti‐GalC:sulfatide complex antibodies were IgG subtype and were detected in one patient with influenza A and *M. pneumoniae* infections. For patients with anti‐GM1 IgG antibodies, 40% (23/58) had cross‐reaction with GM1:GalC complex and 60% solely bound to GM1. For patients with anti‐GM1:GalC complex IgG antibodies, 26% (9/34) showed complex‐independent, 24% (8/34) showed complex‐enhancement, 18% (6/34) showed complex‐attenuated, and 32% (11/34) showed complex‐dependent. For patients with anti‐GM1:sulfatide complex IgG antibodies, 43% (13/30) showed complex‐independent, 13% (4/30) showed complex‐enhancement, 17% (5/30) showed complex‐attenuated, and 27% (8/30) showed complex‐dependent.

## Discussion

We hereby demonstrated that *C. jejuni*, influenza A, influenza B, and hepatitis A virus currently served as the most common cause of antecedent infections in GBS in the Southwest of Shandong Province, Northern China. The proportion of patients following infections of CMV, EBV, and *M. pneumoniae* only accounted for less than 5% of the total patients, respectively. Infections of dengue virus, herpes simplex virus, varicella‐zoster virus, and rubella virus were also detected but only in a minority of patients and not higher than in controls. The proportion of patients with *C. jejuni* infection in our region (27%) was similar to those patients reported in Dutch (32%)^3^ and the UK(26%)^16^ but lower than those reported in Bangladesh (57%)^17^ and a previous study from Northern China (66%).^6^ From 2000 to 2018, the average life expectancy locally increased from 73.9 to 78.1 (unpublished data). The changes in the proportion of GBS patients with *C. jejuni* infection in China may reflect the improved healthy conditions of this country and be related to the rapid development of society and the economy.^18^ The *C*. *jejuni* infection has a strong relation with axonal GBS.^6^, ^17^ Reduced proportion of patients with *C. jejuni* infection may contribute to the predominant GBS subtype in China changed from axonal GBS in the 1990s to demyelinating GBS in 2010s. Notably, seven patients with *C. jejuni* infection complained of upper respiratory tract symptoms. The infection of *C. jejuni* may breakdown the host immune balance and increase the host susceptibility to the infectious diseases.^19^ It is possible that *C. jejuni* infection may cause other infections beyond our study, which accounts for the upper respiratory tract symptoms in the patients.

Patients with GBS in our cohort study displayed infection‐related clinical features. Being similar to the Dutch study,^3^ patients with *C. jejuni* infection had more often complaints of diarrhea than other patients. Although a cohort study from Bangladesh showed no association between the age of patients with GBS and *C. jejuni* infection,^17^ our study showed that *C. jejuni* infection was more frequent in younger patients with GBS, which may reflect a special dietary construct increasing the risk of *C. jejuni* infection in the youth population. Similarly, a recent international study in GBS showed that patients with the axonal subtype are younger than other patients.^2^ However, in our study, there was no difference in frequency of electrophysiological subtypes between patients with *C. jejuni* and other infections. Notably, GBS patients following influenza B infections displayed pure motor deficits and high risk of needing mechanical ventilation (2/7, 29%) while GBS patients following influenza A infections had no need for mechanical ventilation (0/11) and relatively low frequency of patients with GBS‐DS ≥ 4 at nadir. Our study supported influenza B‐related GBS as a severe phenotype of pure motor deficits while influenza A‐related GBS mainly presents as a mild clinical phenotype. Our results were similar to a previous study that none of influenza A‐related GBS (0/8) needed mechanical ventilation while half of the GBS patients following influenza B infection (2/4) needed mechanical ventilation.^20^ The exposure to different types of preceding infection in combination with host factors among different regional or ethnic groups may result in the geographical clinical heterogeneity in GBS worldwide.

Previously, the relation between *C. jejuni* infection and antibodies against gangliosides, including GM1, GM1b, GD1a, GalNAc‐GD1a, and GQ1b, has been well established in GBS.^21^, ^22^ In our region, there was a strong correlation between *C. jejuni* infection and anti‐GM1, anti‐GalNAc‐GD1a, and anti‐GM1:GalC complex antibodies. The gene polymorphism of *cst‐II*, encoding a sialyltransferase in *C. jejuni*, leads to the biosynthesis of different gangliosides‐mimicking LOS.^22^, ^23^ *C. jejuni* strains with *cst‐II* (Asn51) regularly express the GQ1b‐like LOS while the strains with *cst‐II* (Thr51) express more GM1‐like and GD1a‐like LOS.^22^ Typically, IgG antibodies against GQ1b were associated with MFS while the IgG antibodies against GM1 and GD1a were associated with axonal GBS.^24^, ^25^ High incidence of MFS was previously reported in East Asia, especially Japan (up to 26%);^2^, ^26^ however, there was much lower frequency of MFS in both our current study (3%) and a large multicenter study from Southern China (12%).^8^ Our results supported *C. jejuni* with *cst‐II* (Thr51), bearing GM1‐like LOS, as one of the causative pathogens in our region, which deserves a further study using *C*. *jejuni* samples from the patients. Moreover, none of the patients in our cohort were detected with *H. influenzae* infection, which was frequently seen in patients with MFS.^27^ The regional infectious spectrum may partly explain why there was a low frequency of MFS in China.

Liver injury in patients following influenza A and B infections has been described in both the cohort study and case report.^28^, ^29^ Some GBS‐related pathogens including EBV, varicella‐zoster virus, hepatitis A virus, and hepatitis E virus were able to trigger the autoimmune response against liver.^30^, ^31^, ^32^ Although pretreatment liver dysfunction has been reported for a long time,^9^ it remains unknown that the pretreatment liver dysfunction in GBS was caused by antecedent infections or GBS itself. In this study, 13% (17/132) of the patients were detected with pretreatment liver dysfunction at entry. Although the difference in frequency of pretreatment liver dysfunction among patients with and without infections was not significant, GBS patients following influenza B virus infection displayed more susceptibility to pretreatment liver dysfunction. Our study supported antecedent infection as one cause of pretreatment liver dysfunction in GBS patients. However, 19% of the patients without infection also had pretreatment liver dysfunction. It cannot be excluded that some other pathogens beyond our study were associated with liver dysfunction in patients with GBS. None of the GBS patients with *C. jejuni* infection had pretreatment liver dysfunction, which needs further confirmation by other cohort studies.

In conclusion, this is the first study to report the antecedent infectious spectrum in patients with GBS in China. We firstly reported influenza B‐related GBS as a pure motor phenotype. *C*. *jejuni* serves as the predominant cause of antecedent infections in patients with GBS in our region, but the frequency is much lower than 30 years ago. The regional infectious spectrum contributed to the clinical heterogeneity of GBS. Our results deserve further confirmation by other larger cohort studies.

## Author Contributions

Y. Wang developed the concept and design of the article. Y. Hao, W. Wang and B. Qiao and D. Liu performed the experiment. W. Wang and X. Feng collected the clinical data. M. Chen and Y. Wang analyzed all of the data. Y. Hao and Y. Wang wrote the first draft, B.C. Jacobs and Y. Wang performed the critical revision and all the authors critically evaluated the manuscript.

## Conflict of Interest

The authors report no disclosures relevant to the manuscript.

## Supporting information


**Table S1.** Details of ELISA kits of antecedent infections assay.
**Table S2.** Urban and rural distribution of antecedent infections in patients with Guillain‐Barré syndrome.
**Table S3.** Antecedent infections and antibodies to glycolipids and glycolipid complex in patients with Guillain‐Barré syndrome.
