# Supplementary material for: Antecedent infections in Guillain‐Barré syndrome: a single‐center, prospective study
Source: Ann Clin Transl Neurol. 2019 Nov 12;6(12):2510–7. doi: 10.1002/acn3.50946 (PMC6917331; doi:10.1002/acn3.50946)
Supplement: Supplementary file 1 — Table S1. Details of ELISA kits of antecedent infections assay. Table S2. Urban and rural distribution of antecedent infections in patients with Guillain‐Barré syndrome. Table S3. Antecedent infections and antibodies to glycolipids and glycolipid complex in patients with Guillain‐Barré syndrome. [file ACN3-6-2510-s001.docx]

*Research Article, Annals of Clinical and Translational Neurology*

**Antecedent infections in Guillain-Barré syndrome: a single center, prospective study**

Yanlei Hao^1^, MD, Weifang Wang^1^, MM, Bart C Jacobs^2^, MD, Baojun Qiao^1^, MM, Mengshi Chen^3^, MD, Daiqiang Liu^1^, MM, Xungang Feng^1^, MD, Yuzhong Wang^1,4,*^, MD

^1^Department of Neurology, Affiliated Hospital of Jining Medical University, Jining, Shandong Province, China; ^2^Department of Neurology and Immunology, Erasmus University Medical Centre, Rotterdam, The Netherlands; ^3^Department of Epidemiology and Health Statistics, School of Public Health, Central South University, Changsha, Hunan Province, China; ^4^Central laboratory, Affiliated Hospital of Jining Medical University, Jining, Shandong Province, China.

Supplement table 1. Details of ELISA kits of antecedent infections assay

|  | Ig type | Sources of ELISA Kits |
| --- | --- | --- |
| *Campylobacter Jejuni* | IgA, IgM and IgG | Virion and Serion (Würzburg, Germany) |
| Varicella-Zoster Virus | IgM | Virion and Serion (Würzburg, Germany) |
| Herpes simplex virus | IgM | Virion and Serion (Würzburg, Germany) |
| Influenza A and B viruses | IgA, IgM and IgG | Virion and Serion (Würzburg, Germany) |
| Dengue virus | IgM | Virion and Serion (Würzburg, Germany) |
| *Haemophilus influenza* | IgG | DRG Instruments GmbH (Marburg Germany) |
| *Mycoplasma pneumonia* | IgM | Savyon Diagnostics Ltd (Ashdod, Israel) |
| Zika virus | IgM | EUROIMMUN (Lebeck, Germany) |
| Epstein-Barr virus | IgM | Yahuilong Biotechnology (Shenzhen, China) |
| Cytomegalovirus | IgM | Yuande Bio-Medical Engineering (Beijing, China) |
| Rubella virus | IgM | Yuande Bio-Medical Engineering (Beijing, China) |
| Hepatitis A virus | IgM | InTec Products (Xiamen, China) |
| Hepatitis E virus | IgM | Kehua Bio-engineering (Shanghai, China) |

| Supplement table 2. Urban and rural distribution of antecedent infections in patients with Guillain-Barré syndrome | | | | |
| --- | --- | --- | --- | --- |
|  | Urban (n = 30) | Rural (n = 120) | OR (95% CI) | *p* value |
| No infection | 14 (47) | 56 (47) | Reference |  |
| *Campylobacter jejuni* | 6 (20) | 34 (28) | 0.7 (0.3, 1.8) | 0.475 |
| Influenza A | 6 (20) | 20 (17) | 1.2 (0.4, 3.3) | 0.72 |
| Influenza B | 5 (17) | 19 (16) | 1.1 (0.4, 3.0) | 0.925 |
| Hepatitis A virus | 1 (3) | 6 (5) | 0.7 (0.1, 5.8) | 0.712 |
| Dengue virus | 0 (0) | 4 (3) |  |  |
| Cytomegalovirus | 1 (3) | 3 (3) |  |  |
| Epstein-Barr virus | 0 (0) | 4 (3) |  |  |
| *Mycoplasma pneumoniae* | 1 (3) | 2 (2) |  |  |
| Herpes simplex virus | 0 (0) | 3 (3) |  |  |
| Varicella-zoster virus | 1 (3) | 2 (2) |  |  |
| Rubella virus | 0 (0) | 1 (1) |  |  |
| The data were shown as n (%). OR = odds ratio; CI = confidence interval. No infection of hepatitis E virus, *Haemophilus influenzae* and Zika virus were detected. | | | | |

| Supplement table 3. Antecedent infections and antibodies to glycolipids and glycolipid complex in patients with Guillain-Barré syndrome | | | | | |
| --- | --- | --- | --- | --- | --- |
| IgG or IgM antibody to | Total (n = 150) | No infection (n = 70) | *Campylobacter jejuni* (n =25) | Influenza A (n = 11) | Influenza B (n = 7) |
| GM1 | 58 (39) | 23 (33) | 17 (68)* | 1 (9) | 2 (29) |
|  |  |  |  |  |  |
| GM1b | 1 (1) | 0 (0) | 0 (0) | 0 (0) | 0 (0) |
|  | 0 (0) | 0 (0) | 0 (0) | 1 (9) | 0 (0) |
| GM2 |  |  |  |  |  |
|  |  |  |  |  |  |
| GD1a | 16 (11) | 5 (7) | 5 (20) | 0 (0) | 1 (14) |
|  |  |  |  |  |  |
| GD1b | 29 (19) | 10 (14) | 8 (32) | 1 (9) | 2 (29) |
|  |  |  |  |  |  |
| GalNAc-GD1a | 16 (11) | 3 (4) | 9 (36)* | 1 (9) | 0 (0) |
|  |  |  |  |  |  |
| GQ1b | 20 (13) | 12 (17) | 5 (20) | 1 (9) | 1 (14) |
|  |  |  |  |  |  |
| GM1:GalC | 34 (23) | 14 (20) | 11 (44)* | 1 (9) | 1 (14) |
|  |  |  |  |  |  |
| GM1:sulfatide | 30 (20) | 13 (19) | 10 (40) | 3 (27) | 2 (29) |
|  |  |  |  |  |  |
| GalC:sulfatide | 1 (1) | 0 (0) | 0 (0) | 0 (0) | 0 (0) |
| All data were shown as n (%). Neither IgG nor IgM antibodies against GM3, GT1a, GalC, sulfatide and GalC:cholesterol complex were detected. **p*<0.05, vs no infection, chi-square test. | | | | | |
